# Hospital Meetings, &c.

**Published:** 1899-04-15

**Authors:** 


### HOSPITAL MEETINGS, &C.

### NORTH-WEST LONDON HOSPITAL.

Mr. George Herring, the treasurer, presided at the twentieth annual court of governors of the North-West London Hospital on Monday last, 10th inst. From the report presented it appeared that the income for the year was £8,387, £3,180 of this being ordinary, £5,107 a special donation, and £100 from legacies. The special donation was from Mr. Herring, to compensate for last year's disappointment in reference to the Prince of Wales's Fund and for the falling-off of income during the Diamond Jubilee year. The total expenditure amounted to £4,284, including £27 on building improvements. The annual subscriptions totalled £714, and the year's income also included £300 from the Hospital Sunday Fund, £128 from the Saturday Fund, £489 proceeds of a bazaar, and £300 from the Prince's Fund. The committee were gratified by the complimentary terms in which the visitors from the last-named body spoke of the work carried on, which, in their opinion, deserved a more commodious and better building. One of these gentlemen sent a Christmas-box of £10 as a mark of his appreciation. The report concluded with an appeal for continued and increased help. The Chairman said he thought the fact that they had treated 601 in-patients, and that there had been over 40,000 attendances in the out-patients' department, showed the necessity for a hospital in that locality, while the approval of the visitors appointed by the Prince's Fund showed that it was conducted on proper lines. As a matter of fact they could compare with any other hospital in the metropolis. The cost of each in-patient was only 22s. per week, while the out-patients cost only 1s. 11d. each. He did not think any other charity could show so little spent on administration. The report was adopted, and cordial votes of thanks to the medical officers and to the ladies concluded the proceedings.

### MILLER HOSPITAL AND ROYAL KENT DISPENSARY.

The annual general meeting of the Miller Hospital and Royal Kent Dispensary was held in the boardroom of the institution, Greenwich Road, on Monday evening, April 10th, under the presidency of Mr. Edgar Sydney. The report of the committee, which was read by Mr. James Marks, the secretary, stated that they were able to show a balance of income over expenditure for the year 1898, which satisfactory position was due to the receipt of legacies, one of £50, another of £100, and a third of £2,000. There had been an increase in annual subscriptions of £62, and new subscriptions amounting to £98. The sum of £552 was received in donations. The Amalgamated Friendly Societies of Lewisham had placed its donation to the Extension Fund, and the Skinners' Company also contributed £50 to the same fund. This the committee say was most acceptable, as only a very small portion of the sum originally appealed for has been received. In addition to the necessity for extra hospital accommodation, other important alterations are pressing, viz., the erection of isolation wards and alterations and improvements in the nurses' quarters. The committee express their great obligation to the members of the Blackheath Amateur Operatic Society, which has contributed by its various performances sums aggregating £950. Other donations received were £200 from the Prince of Wales's Fund, £202 from the Metropolitan Sunday Fund, and £66 from the Saturday Fund. The work of the institution is still increasing, 18,410 patients having been treated during the year, this being an increase of 962 over the previous year. The accounts presented showed that the total ordinary income for the year was £2,902, and the extraordinary income to have amounted to £2,150, together £5,052. The total ordinary expenditure was £3,492, and the extraordinary expenditure £15, which, with investments on life subscriptions, £52, leaves a balance of income over expenditure amounting to £1,493. The Chairman, in moving the adoption of the report, expressed the pleasure the committee felt that they had a balance on the right side of the ledger. Not for many years had they been able to experience the satisfaction of finding their income exceed their expenditure. This position was due to munificent legacies—one for £2,000 from the executors of the late Mr. Francis Gilbert—and it had enabled the committee to avoid resorting to the bank, or having to sell stock, which they could ill afford to do. They hoped subscriptions would continue to come in, for they very much desired to carry out the extension, as they had to turn away applicants for relief for want of room. They were also desirous of getting the isolation wards and the improvements needed in the nurses' quarters. The Chairman concluded by warmly eulogising the medical staff and officers, and paid a tribute to the interest taken in the institution by the Rev. Brook Lambert and Mr. Howard, the hon. solicitor to the hospital. Mr. W. Taylor seconded the motion for the adoption of the report, which was unanimously agreed to. The committee having been re-elected, and Sir John Knill appointed a trustee in the room of his late father, Sir Stuart Knill, the proceedings terminated with the passing of votes of thanks to the medical staff, the committee, and to the chairman for presiding.

